# One‐year safety, healing and amputation rates of Wagner 3‐4 diabetic foot ulcers treated with cryopreserved umbilical cord (TTAX01)

**DOI:** 10.1111/wrr.12809

**Published:** 2020-05-09

**Authors:** William A. Marston, John C. Lantis, Stephanie C. Wu, Aksone Nouvong, John Randolph Clements, Tommy D. Lee, Nicholas D. McCoy, Herbert B. Slade, Scheffer C. Tseng

**Affiliations:** ^1^ Division of Vascular Surgery University of North Carolina School of Medicine Chapel Hill North Carolina USA; ^2^ Mt Sinai West and St Luke's Hospitals New York New York USA; ^3^ Rosalind Franklin University North Chicago Illinois USA; ^4^ Department of Surgery UCLA Los Angeles California USA; ^5^ Carilion Clinic Roanoke Virginia USA; ^6^ TissueTech, Inc. Miami Florida USA; ^7^ Department of Pediatrics University of North Texas Health Sciences Center Fort Worth Texas USA

## Abstract

An open label, multicenter 16‐week trial of cryopreserved human umbilical cord (TTAX01) was previously undertaken in 32 subjects presenting with a Wagner grade 3 or 4 diabetic foot ulcer, with 16 (50%) of these having confirmed closure following a median of one product application (previous study). All but two subjects (30/32; 94%) consented to participate in this follow‐up study to 1‐year postexposure. No restrictions were placed on treatments for open wounds. At 8‐week intervals, subjects were evaluated for adverse events (AEs) and wound status (open or closed). Average time from initial exposure to end of follow‐up was 378 days (range 343‐433), with 29 of 30 (97%) subjects completing a full year. AEs were all typical for the population under study, and none were attributed to prior exposure to TTAX01. One previously healed wound re‐opened, one previously unconfirmed closed wound remained healed, and nine new wound closures occurred, giving 25 of 29 (86.2%) healed in the ITT population. Three of the new closures followed the use of various tissue‐based products. Three subjects whose wounds were healed required subsequent minor amputations due to osteomyelitis, one of which progressed to a major amputation (1/29; 3.4%). One additional subject underwent two minor amputations prior to healing. Overall, the study found TTAX01 to be safe in long‐term follow‐up and associated with both a low rate of major amputation and a higher than expected rates of healing.

## 1 INTRODUCTION

People with diabetes mellitus are at substantial risk of developing a foot ulcer at some point in their lifetime. Unlike acute cutaneous injuries in otherwise healthy individuals, diabetic foot ulcers (DFU) typically exhibit delayed healing, a downstream consequence of prolonged hyperglycemia, which triggers a wide range of molecular events leading to neural and vascular damage.^1^ Neuropathy increases the risk of ulcer formation, compounded by alterations in molecular and cellular responses to wounding including a dysregulated inflammatory response which invites poorly controlled local infection and chronicity.^2^, ^3^, ^4^ Chronically unhealed and infected DFU can eventually lead to the need for minor or major lower extremity amputation.^5^, ^6^ In fact, DFU is the most common single precursor of lower extremity amputations among persons with diabetes, whose age‐adjusted amputation rate is nearly 30 times that of people without diabetes.^7^

The short‐term goals of treatment of DFU complicated by osteomyelitis, but not ischemia, are to remove devitalized bone and tissue, identify and treat infection, and achieve wound healing with the least disruption to anatomy. The longer term goals are maintainence of healing over a meaningful length of time, and preservation of the limb, each of which is challenging given historically high rates of ulcer recurrence and amputation.^8^

The present study was designed as an observational extension study from a previous interventional trial^9^ of TTAX01, an aseptically processed cryopreserved human umbilical cord product derived from donated human placental tissue following healthy, live, caesarian section, full‐term births, after determination of donor eligibility and placenta suitability.

The regulation of birth tissue products used in the treatment of DFU is undergoing a transition from the application of a combined set of rules (US Public Health Service Act §361, US FDA regulations 21 CFR §1271) to the application of rules governing tissue based biological products.^10^ The shift to regulating these products as biologics requires manufacturers to undertake well‐designed clinical evaluations of safety and efficacy under an investigational new drug exemption, in order to support a biologics license application. With the expectation of needing to treat recurrent ulcers over substantial periods of time, follow‐up studies of 12‐months duration are mandated by the FDA for the purpose of evaluating long‐term safety.

## 2 METHODS AND PATIENTS

### 2.1 Trial design and participants

This was a multicenter, noninterventional follow‐up study conducted at healthcare facilities in the United States (11 centers), where each center had previously enrolled at least one patient into a preceding interventional trial of TTAX01 for the treatment of Wagner 3‐4 DFU (NCT03230175).^9^ The protocol received Institutional Review Board approval for each participating center. Most subjects from the preceding trial consented to participate and enrolled into this follow‐up (30/32; 94%) based on the only inclusion criteria that they participated in the preceding trial and were exposed to at least one application of TTAX01. Subjects enrolled into this study upon completion of the preceding trial, which occurred after confirmation of healing at any point, or upon exiting that 16‐week trial with an unhealed wound. No restrictions were placed on surgical interventions, offloading techniques or the use of any therapies for wounds that were still open at the time of enrollment into this study. Visits were scheduled at 8 week intervals to complete at least 52 weeks from the time of enrollment into the preceding trial. All enrolled subjects (n = 30) were evaluable for safety, while subjects with a remaining open or closed index wound at enrollment (n = 29) were evaluable for efficacy.

### 2.2 Evaluations

Safety was evaluated by frequency, expectedness, and relationship of adverse events (AEs) calculated for each body system, by preferred terminology, by the preceding treatment, for number of subjects and percentage reporting the event. Wounds were evaluated using the eKare inSight measuring device (eKare Inc., Fairfax, Virginia) by capturing an image for electronic measurement via automatic tracing of area (cm^2^), depth (cm), and volume (cm^3^). Wound status (healed, not healed) was recorded at each visit. New wound closures were recorded based on a single observation of closure rather than the more conservative regulatory requirement of closure confirmation at two subsequent visits each 2 weeks apart, which was followed during the preceding treatment study. Subjects who achieved closure and remained closed at the final visit were considered to have healed their wound.

### 2.3 Endpoints

The primary objective of the study was to identify new AEs and examine ongoing AEs not resolved in subjects who were exposed to TTAX01 in the TTCRNE‐1501 trial. The primary efficacy endpoint was a categorical determination of complete wound healing of the index wound determined by either a single opinion of the Investigator in cases where the wound remained open (healed = no), a concordance of opinion between the Investigator and a single independent expert reviewer of the eKare images in cases where the Investigator reported the wound as healed (healed = yes), or the opinion of a second independent “tie breaker” reviewer in the event that the Investigator and first reviewer had discordant opinions. The primary efficacy calculation was performed for the modified ITT (mITT) population.

AEs, including index wound AEs and systemic AEs, were coded for preferred terminologies using Medical Dictionary for Regulatory Activities (MedDRA). AE were grouped into pre‐enrollment and post‐enrollment categories based on the enrollment date and event start date. New and ongoing therapies for the index wound were reported.

### 2.4 Statistical Analysis

All continuous data were expressed as mean ± SD (range), while categorical variables were expressed as frequency and percentages. The primary efficacy analysis was performed for the mITT population, which included all enrolled subjects having a wound (healed or open) at baseline.

## 3 RESULTS

Demographics and baseline characteristics for all 30 subjects are summarized in Table 1. Details on the full cohort enrolled in the previous trial have been published.^9^ Twenty‐nine (97%) of 30 enrolled subjects were followed for 12 or more months from initial exposure. One subject was lost to follow‐up at 48 weeks, having achieved wound closure at 32 weeks. All 30 were evaluable for safety, but only 29 were evaluable for efficacy as one subject had his unhealed wound removed in a minor amputation in the previous study.

**TABLE 1:** Demographics and baseline characteristics

	N = 30
Age	57.1 ± 9.9 (41.0‐72.0)
Gender	23 (76.7%)
Female	1 (3.3%)
Male	29 (96.7%)
Race	
Alaskan Native/AI	1 (3.3%)
Black/African American	8 (26.7%)
White/Caucasian	20 (66.7%)
Other	1 (3.3%)
Ethnicity	
Hispanic/Latino	7 (23.3%)
Not Hispanic/Latino	23 (76.7%)
Minor amputation in prior study	16 (53.3%)

*Note:* Values are reported as mean ± SD (min–max) or number (%).

Twenty‐four (80%) of 30 subjects experienced one or more AE during the study. All AEs were expected within the demographic although not necessarily expected for individual subjects. None of them was related to prior exposure to TTAX01. Nine subjects experienced one or more serious AE, defined as requiring hospitalization; none of these events was considered related to prior exposure to TTAX01. There were no deaths.

Four subjects (13%) of 30 underwent one or more minor (below the ankle) amputations, with one of them (3.3%) eventually undergoing a major below the knee amputation. That subject was one of 17 who had undergone minor amputation at the initial baseline surgical resection in the previous trial, giving a rate of 1 of 17 (5.9%) for progression from minor to major amputation across both studies. Three subjects had healed their target ulcer prior to the amputations, while one had minor amputations unrelated to the target ulcer, which eventually healed. It was an intriguing observation that cutaneous healing was not always indicative of resolution of underlying infection, as seen in the cases where the wound healed but surgical removal of bone and tissue was later required due to osteomyelitis, which was either ongoing or recurrent.

There were no disagreements between the Investigator and the expert reviewer regarding final wound status. A high proportion of subjects maintained or achieved persistent closure of their index wound by the end of this long‐term follow‐up study. One of the 16 previously confirmed closed wounds re‐opened. One wound that was previously coded as open, simply because of missing a confirmation of closure visit in the previous treatment study, enrolled into this study with the wound closed, giving 16 with closed wounds at the start of this follow‐up. Fifteen (93.8%) of these subjects experienced a persistent closure through 1 year from initial treatment. Across the two studies, 27 wounds achieved initial closure, one of which failed confirmation of closure and remained open, and one of which was confirmed closed but re‐opened during the observational period, giving 25 persistently closed wounds.

**FIGURE 1:**
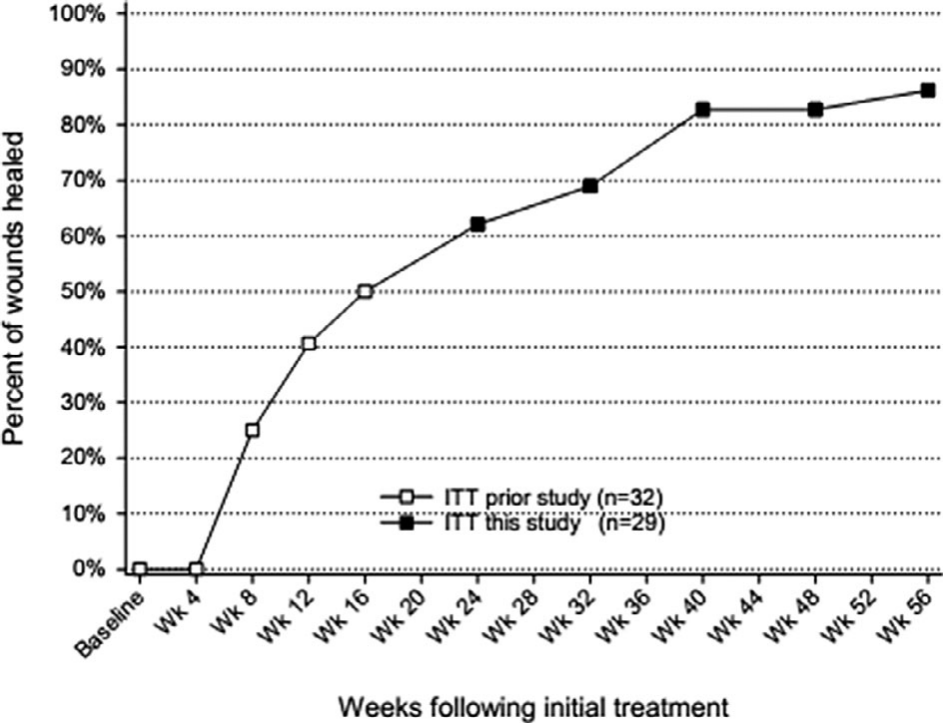
Wound closures occurring in the prior study (□) and the present (■) 1 year follow‐up

**FIGURE 2:**
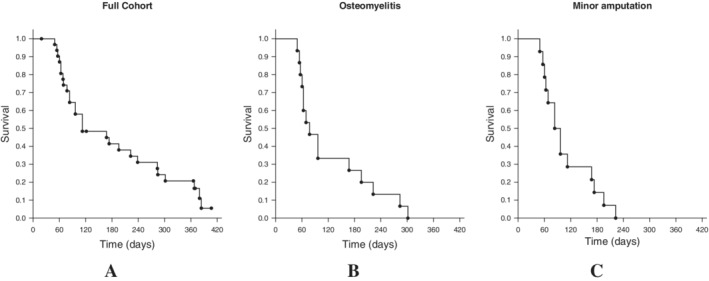
Median time to heal. A, Full cohort (112 days, range 49‐383), B, those with confirmed osteomyelitis who went on to heal (77 days, range 49‐301); and, **C,** those with baseline minor amputations who went on to heal (83 days, range 49‐222). Some points represent more than one subject

Nine subjects who had not achieved confirmed healing in the prior study had a final evaluation of wound closure in this study (Table 2). Among the 29 subjects with evaluable wounds, 25 (86.2%) ended this follow‐up study with a final determination of wound closure (Figure 1). Median times to closure for all subjects, and those who healed despite baseline osteomyelitis or minor amputation are given in Figure 2.

**TABLE 2:** Proportion of wounds closed, by visit. Wound closure in the prior study was confirmed at two visits, 2 weeks apart. In this follow‐up study, confirmatory visits were not used

	Prior Study	Present Study	FUV 1 Wk 24	FUV2 Wk32	FUV3 Wk40	FUV4 Wk48	FUV5 Wk56
n	16	n	18	20	24	24	25
ITT	32	mITT	29	29	29	29	29
Proportion (%)	50.0		62.1	69.0	82.8	82.8	86.2

Abbreviations: ITT, Intent to Treat population; mITT, modified Intent to Treat; n, number of subjects with observed wound closure.

Among 20 with biopsy confirmed osteomyelitis at baseline in the previous study, 16 (80%) achieved confirmed closure, and 15 (75%) maintained persistent closure, as did 14 (87.5%) of 16 who had undergone a baseline minor amputation in the prior study and enrolled into this follow‐up. At the intersection of these subsets, 10 (90.9%) of 11 subjects with both confirmed osteomyelitis and a baseline minor amputation went on to achieve complete wound closure across the two studies.

Among the nine who achieved closure in follow‐up, three required no advanced therapies, three were treated with silver‐based products in addition to standard care, and three were treated with tissue‐based products (NEOX × 1, or Kerecis × 5, or Apligraf × 4 + EpiFix × 5) before closing. One subject with a persistently open wound received negative pressure wound therapy, without achieving closure, while another received a different amniotic tissue product (Affinity) without success.

## 4 DISCUSSION

The findings in this multicenter, long‐term follow‐up study are encouraging. No safety signals were detected with respect to prior exposure to the human cryopreserved umbilical cord product (TTAX01), with AEs being unrelated and typical for the population under study.

Not only were healing rates high in the previous and present follow‐up study, healing times among subjects with minor baseline amputations in the previous study were much shorter than those reported by Svensson et al, who saw a median time from primary amputation to wound healing of 26 weeks (range 2‐250).^11^ They further reported that 21% of these patients required re‐amputation above the ankle, 3.5 times higher than in the present study.

Published experience in treating DFU with a range of amnion, chorion and umbilical cord products has been universally positive, although the majority of studies have been with shallow Wagner 1 ulcers, which have no exposure of muscle, fascia, joint capsule or bone.^12^, ^13^ Significant differences in the proportion of wounds healed in comparison to standardized care were often seen as early as 4 weeks from initiation of therapy. One dehydrated amnion and chorion product gave remarkably positive but inconsistent results over a series of single center, cross‐over, multicenter and multicenter extension trials, with wound closure rates of 90 to 100% seen by 6 to 12 weeks.^14^, ^15^, ^16^, ^17^, ^18^, ^19^

A cryopreserved product showed superiority over standard care in published studies of Wagner 1 DFU, with efficacy over 12 weeks in the range of 62 to 71% in both prospective and retrospective studies.^20^, ^21^, ^22^ A large confirmatory study (NCT02571738) was abandoned however, after approximately 50% of subjects had enrolled.

One trial of a dehydrated umbilical cord product^23^ showed lower healing rates than were seen with a dehydrated amnion and chorion product from the same manufacturer, although it was still superior to standardized care.

Wound recurrence rates have not been routinely reported. Publications where recurrence is mentioned give rates of 14%^24^ and 18%^20^ at 3 months of follow‐up with two different products. A third product reported on 82% of healed wounds followed for 9 to 12 months post healing, at which time 94% remained healed.^8^ Although the present study reports follow‐up over 1 year from initial exposure, rather than from the time of initial healing, persistent healing was seen in over 90% of wounds that initially healed. Of course, this study is without a control arm, therefore we do not know the freedom from recurrence rates for patients who would have healed without advanced therapy.

The patients who enrolled in the prior interventional study, TTCRNE‐1501, presented with high grade Wagner 3‐4 DFU which literature and database searches reveal to be particularly difficult to heal. The achievement of a very high rate of healing over the course of this and the previous trial is unexpected, and requires confirmation. Another unexpected finding was the discordance between the primary efficacy endpoint of a healed cutaneous wound, and the expected clinical benefit of a healed foot. Surprisingly, three subjects who achieved and maintained cutaneous healing ended up requiring various degrees of amputation during follow‐up as a result of recurrent or persistent osteomyelitis.

It is important to acknowledge that the previous interventional study was open label without concurrent control, and that additional therapies during this follow‐up were unrestricted. In particular, the benefit of prior TTAX01 treatment is unknown in the three subjects who healed following use of a tissue‐based product during this observational study. Consequently, it remains to be seen whether the use of TTAX01 in treating high grade, complex DFU may be superior to standard care when studied in a multicenter randomized trial.

Available evidence suggests that standard care alone is unlikely to produce the healing rates seen in this study. In a series of cases of diabetic foot osteomyelitis managed with limb‐sparing surgery, Johnson et al reported that 59% had healed by 1 year,^25^ which was regarded as an improvement over outcomes reported by Nehler et al, in which only 34% of patients achieved complete healing.^26^ In contrast, but consistent with the findings reported here, Caputo et al^27^ reported healing in 79% of 33 Wagner 3‐4 wounds over a period of 1 year, using the commercial product NEOX CORD 1 K. A collaborative review with Healogics, Inc., of real world data for Wagner 3‐4 wounds indicates that healing rates are normally low in this population.

The results of this long‐term follow‐up study continue to be encouraging given the unmet medical need of a treatment for foot wounds complicated with osteomyelitis, in a diabetic population exhibiting high morbidity and mortality. We believe that the healing outcomes and favorable safety data generated in this initial study support proceeding to a prospective multicenter randomized study of TTAX01 in treating Wagner grade 3‐4 wounds.

## References

[1] Park S , Kang HJ , Jeon JH , Kim MJ , Lee IK . Recent advances in the pathogenesis of microvascular complications in diabetes. Arch Pharm Res. 2019;42(3):252‐262. 30771210 10.1007/s12272-019-01130-3

[2] Blakytny R , Jude E . The molecular biology of chronic wounds and delayed healing in diabetes. Diabet Med. 2006;23(6):594‐608. 16759300 10.1111/j.1464-5491.2006.01773.x

[3] Rafehi H , El‐Osta A , Karagiannis TC . Genetic and epigenetic events in diabetic wound healing. Int Wound J. 2011;8(1):12‐21. 21159125 10.1111/j.1742-481X.2010.00745.x PMC7950456

[4] Ratter JM , Tack CJ , Netea MG , Stienstra R . Environmental signals influencing myeloid cell metabolism and function in diabetes. Trends Endocrinol Metabol. 2018;29(7):468‐480. 10.1016/j.tem.2018.04.008 29789206

[5] Singh N , Armstrong DG , Lipsky BA . Preventing foot ulcers in patients with diabetes. JAMA. 2005;293(2):217‐228. 15644549 10.1001/jama.293.2.217

[6] Geiss LS , Li Y , Hora I , Albright A , Rolka D , Gregg EW . Resurgence of diabetes‐related nontraumatic lower‐extremity amputation in the young and middle‐aged adult U.S. population. Diabetes Care. 2019;42(1):50‐54. 30409811 10.2337/dc18-1380

[7] Sen CK , Gordillo GM , Roy S , et al. Human skin wounds: a major and snowballing threat to public health and the economy. Wound Repair Regen. 2009;17(6):763‐771. 19903300 10.1111/j.1524-475X.2009.00543.x PMC2810192

[8] Zelen CM , Serena TE , Fetterolf DE . Dehydrated human amnion/chorion membrane allografts in patients with chronic diabetic foot ulcers: a long‐term follow‐up study. Wound Med. 2014;4:1‐4.

[9] Marston WA , Lantis JC 2nd , Wu SC , et al. An open‐label trial of cryopreserved human umbilical cord in the treatment of complex diabetic foot ulcers complicated by osteomyelitis. Wound Repair Regen. 2019;27(6):680‐686. 31376297 10.1111/wrr.12754 PMC6900178

[10] U. S. Food and Drug Administration . Regulatory Considerations for Human Cell, Tissues, and Cellular and Tissue‐Based Products: Minimal Manipulation and Homologous Use. Guidance for Industry and Food and Drug Administration Staff: U. S. Food and Drug Administration; 2017.

[11] Svensson H , Apelqvist J , Larsson J , Lindholm E , Eneroth M . *Minor* amputation in patients with diabetes mellitus and severe foot ulcers achieves good outcomes. J Wound Care. 2011;20(6):261‐262. 4, 6 passim. 21727875 10.12968/jowc.2011.20.6.261

[12] Laurent I , Astere M , Wang KR , Cheng QF , Li QF . Efficacy and time sensitivity of amniotic membrane treatment in patients with diabetic foot ulcers: a systematic review and meta‐analysis. Diabetes Ther. 2017;8(5):967‐979. 28895073 10.1007/s13300-017-0298-8 PMC5630554

[13] Haugh AM , Witt JG , Hauch A , et al. Amnion membrane in diabetic foot wounds: a meta‐analysis. Plast Reconstr Surg Glob Open. 2017;5(4):e1302. 28507863 10.1097/GOX.0000000000001302 PMC5426882

[14] Zelen CM , Serena TE , Denoziere G , Fetterolf DE . A prospective randomised comparative parallel study of amniotic membrane wound graft in the management of diabetic foot ulcers. Int Wound J. 2013;10(5):502‐507. 23742102 10.1111/iwj.12097 PMC4232235

[15] Zelen CM . An evaluation of dehydrated human amniotic membrane allografts in patients with DFUs. J Wound Care. 2013;22(7):347‐351. 24159656 10.12968/jowc.2013.22.7.347

[16] Zelen CM , Serena TE , Snyder RJ . A prospective, randomised comparative study of weekly versus biweekly application of dehydrated human amnion/chorion membrane allograft in the management of diabetic foot ulcers. Int Wound J. 2014;11(2):122‐128. 10.1111/iwj.12242. 24618401 PMC4235421

[17] Zelen CM , Gould L , Serena TE , Carter MJ , Keller J , Li WW . A prospective, randomised, controlled, multi‐Centre comparative effectiveness study of healing using dehydrated human amnion/chorion membrane allograft, bioengineered skin substitute or standard of care for treatment of chronic lower extremity diabetic ulcers. Int Wound J. 2015;12(6):724‐732. 25424146 10.1111/iwj.12395 PMC7950807

[18] Zelen CM , Serena TE , Gould L , et al. Treatment of chronic diabetic lower extremity ulcers with advanced therapies: a prospective, randomised, controlled, multi‐Centre comparative study examining clinical efficacy and cost. Int Wound J. 2016;13(2):272‐282. 26695998 10.1111/iwj.12566 PMC7949818

[19] Tettelbach W , Cazzell S , Reyzelman AM , Sigal F , Caporusso JM , Agnew PS . A confirmatory study on the efficacy of dehydrated human amnion/chorion membrane dHACM allograft in the management of diabetic foot ulcers: a prospective, multicentre, randomised, controlled study of 110 patients from 14 wound clinics. Int Wound J. 2019;16(1):19‐29. 30136445 10.1111/iwj.12976 PMC7379535

[20] Lavery LA , Fulmer J , Shebetka KA , et al. The efficacy and safety of Grafix([R]) for the treatment of chronic diabetic foot ulcers: results of a multi‐Centre, controlled, randomised, blinded, clinical trial. Int Wound J. 2014;11(5):554‐560. 25048468 10.1111/iwj.12329 PMC7951030

[21] Lavery L , Fulmer J , Shebetka KA , et al. Open‐label extension phase of a chronic diabetic foot ulcer multicenter, controlled, randomized clinical trial using cryopreserved placental membrane. Wounds. 2018;30(9):283‐289. 30256747

[22] Raspovic KM , Wukich DK , Naiman DQ , et al. Effectiveness of viable cryopreserved placental membranes for management of diabetic foot ulcers in a real world setting. Wound Repair Regen. 2018;26(2):213‐220. 29683538 10.1111/wrr.12635

[23] Tettelbach W , Cazzell S , Sigal F , et al. A multicentre prospective randomised controlled comparative parallel study of dehydrated human umbilical cord (EpiCord) allograft for the treatment of diabetic foot ulcers. Int Wound J. 2019;16(1):122‐130. 30246926 10.1111/iwj.13001 PMC7380046

[24] Thompson P , Hanson DS , Langemo D , Anderson J . Comparing human amniotic allograft and standard wound care when using total contact casting in the treatment of patients with diabetic foot ulcers. Adv Skin Wound Care. 2019;32(6):272‐277. 31082818 10.1097/01.ASW.0000557831.78645.85

[25] Johnson MJ , Shumway N , Bivins M , Bessesen MT . Outcomes of limb‐sparing surgery for osteomyelitis in the diabetic foot: importance of the histopathologic margin. Open Forum Infectious Diseases. 2019;6(10):ofz382. 31660346 10.1093/ofid/ofz382 PMC6796992

[26] Nehler MR , Whitehill TA , Bowers SP , et al. Intermediate‐term outcome of primary digit amputations in patients with diabetes mellitus who have forefoot sepsis requiring hospitalization and presumed adequate circulatory status. J Vasc Surg. 1999;30(3):509‐517. 10477644 10.1016/s0741-5214(99)70078-9

[27] Caputo WJ , Vaquero C , Monterosa A , et al. A retrospective study of cryopreserved umbilical cord as an adjunctive therapy to promote the healing of chronic, complex foot ulcers with underlying osteomyelitis. Wound Repair Regen. 2016;24(5):885‐893. 27312890 10.1111/wrr.12456

